# Atrial fibrillation following bendamustine in three lymphoma patients

**DOI:** 10.1177/10781552251369445

**Published:** 2025-08-17

**Authors:** Najwa Al Himali, Khalil Al Farsi

**Affiliations:** 1 Department of Pharmacy, 194179 Sultan Qaboos University Hospital, Muscat, Oman; 2 Department of Hematology, 194179 Sultan Qaboos University Hospital, Muscat, Oman

**Keywords:** Bendamustine, atrial fibrillation, lymphoma, arrhythmia, rate control, rituximab, cardiology

## Abstract

**Introduction:**

Bendamustine is a chemotherapeutic agent combining alkylating activity with purine analogue properties used for a wide range of malignancies including lymphoma. It is not generally associated with cardiac toxicity.

**Case report:**

In this report, we describe 3 cases of patients with marginal zone lymphoma (MZL), diffuse large B-cell lymphomas (DLBCL), and relapsed classic Hodgkin lymphoma (cHL), who developed atrial fibrillation (AF) 1 to 2 days post bendamustine initiation at a dose ranging from 70–90 mg/m^2^.

**Management and outcomes:**

The patients were managed with bisoprolol ± digoxin and anticoagulation. They continue to be in AF and are under routine follow-up by cardiology. The patients’ lymphoma treatment regimens were changed to alternatives except for the last case, in which she was challenged with another subsequent exposure to bendamustine before she passed away.

**Discussion:**

Old age, hypertension (HTN), diabetes, and ischemic heart disease (IHD) were possible risk factors for AF. However, one of the reported cases is a patient without any known risk factors. Further studies are needed to confirm this observation, to identify patients at risk, and to investigate the underlying mechanisms of bendamustine-induced AF.

## Introduction

AF is among the most common types of arrhythmias in humans with a higher incidence among old patients. It has been associated with an increased risk of stroke, peripheral embolism, and death. Risk factors like obesity, HTN, diabetes, coronary artery disease, valvular heart disease, cardiac surgery, hyperthyroidism, and family history of AF may play a role in AF occurrence. Possible elements of AF pathophysiology are atrial fibrosis, oxidative stress by reactive oxygen species, and inflammation.^1,2^

Cancer and medications used to treat cancer increase the risk of AF. Examples of chemotherapy agents known to cause AF are anthracyclines and alkylating agents such as melphalan and cisplatin.^3^ More recently, many clinical trials have shown an association between Bruton's tyrosine kinase inhibitors (like ibrutinib) and a higher incidence of AF.^3–6^

The onset of chemotherapy-induced AF varied among different medications. For example, anthracycline agents can cause AF months (minimum of 7 months) after therapy. The mechanisms for anticancer agents-induced AF are still not fully clear. It is believed that AF can happen due to any abnormalities in impulse formation or electrical conduction. Medications like ibrutinib are believed to cause AF via multiple complex pathways such as through induction of structural remodeling (atrial dilation and fibrosis), through disruption of calcium homeostasis in the atrium, and through the inhibition of cardiac signaling pathways such as PI3K-Akt signaling and C-terminal Src kinase (CSK). However, exact mechanism remains unclear. Agents with delayed onset of AF (like anthracyclines) are believed to affect atrial structure leading to remodeling. Atrial remodeling increases the vulnerability of AF, but it's role as causative role remains unclear. The mechanisms by which other anticancer agents induce AF are still under investigation. Anticancer agents are usually combined with other agents while treating cancer like steroids and immunotherapy. Some of these combinations will result in increased risk for AF. A higher risk for AF is also observed when chemotherapy agents are used concomitantly with some antiemetic agents.^2,3,7,8^

Bendamustine is a widely used alkylating agent acting via two mechanisms. It has a P53-dependent alkylating agent, inducing apoptosis, and it downregulates mitotic checkpoints thereby inducing mitotic catastrophe. Its DNA damage is more potent compared to other alkylating agents. It possesses activity against various types of human cancers. It is approved by the US Food and Drug Administration and the European Medicines Agency for the management of indolent and aggressive B cell non-Hodgkin lymphoma (NHL), chronic lymphocytic leukemia (CLL), Hodgkin lymphoma (HL), and multiple myeloma (MM).^9,10^

Bendamustine can cause different types of toxicities, but is generally not associated with cardiac toxicity especially AF. Hematological and gastrointestinal adverse effects (AEs) are common with bendamustine use, and they are usually mild to moderate in severity. Data from 9-year post-marketing pharmacovigilance reported several AEs with the use of bendamustine in 3679 patients, 2441 of whom experienced unexpected and serious AEs. Among the reported serious AEs is AF, with eighty reports worldwide.^11,12^ Reporting bendamustine-associated AEs (especially less frequently reported ones) in a larger and more heterogeneous population would help in better understanding its safety profile. In this report, we present 3 patients with different lymphoma subtype who developed AF following bendamustine administration.

## Cases presentations

### Case 1. A patient with MZL who developed AF post-cycle 1 bendamustine-rituximab (BR)

A 49-year-old woman, who was not known to have any comorbidities, was diagnosed with nodal MZL with bulky disease (in the neck) but no evidence of transformation. She was referred for radiotherapy but the extent of disease was believed to be locally extensive and she was advised to start chemotherapy. She was started on BR (bendamustine 90 mg/m^2^ and rituximab 375 mg/m^2^). Two days post-chemotherapy, she presented to the emergency with shivering and vomiting. She had two episodes of hypotension with blood pressure (BP) of 92/57 and 95/59 mmHg. Her baseline BP was 140/90. A 12-lead electrocardiogram (ECG) showed new onset AF with a ventricular rate of 142 beats per minute (bpm). She had no evidence of infection, electrolyte abnormalities, thyroid disturbance, coronary artery disease/ischemia, valvular heart disease, pulmonary embolism or other causes. Ejection fraction (EF) was normal.

The patient was seen by the cardiology team and classified as low risk (CHADSVASC score was only 1) not requiring anticoagulation. Bisoprolol 5 mg and amiodarone 300 mg over 1 h followed by 900 mg for 24 h (for 1 day) were started. She converted to normal sinus rhythm. Bisoprolol was continued without amiodarone. She had a good response to one cycle of chemotherapy and treatment was changed to involved site chemotherapy (ISRT). Three months post-ISRT, PET-CT scan showed a complete metabolic response.

### Case 2. Af diagnosed in a patient with DLBCL post a single cycle of BR (bendamustine and rituximab)

The second patient was an 81-year-old man with type 2 diabetes mellitus, HTN, hypothyroidism, coronary artery disease (with a history of percutaneous coronary intervention), renal insufficiency (CKD stage 3), Alzheimer's disease, and a history of prostate surgery. This patient presented with more than six months history of dysphagia and was diagnosed as DLBCL (with background of low-grade NHL). Staging PET-CT scan showed advanced stage disease. EF was low on 2D-echocardiography (ECHO) (less than 40%). He was treated with BR (bendamustine 70 mg/m^2^ and rituximab 375 mg/m^2^). Two days after cycle 1, the patient presented to the hospital with an impaired level of consciousness and generalized weakness. He was tachycardiac with a heart rate (HR) of 160 bpm and hypotensive (BP 98/57 mmHg). CT-brain did not show any evidence of acute stroke. ECG showed AF with a fast ventricular response (prior ECG showed sinus rhythm), and he underwent chemical cardioversion with amiodarone along with enoxaparin as an anticoagulant. He had a high CHADSVASC score of 4 and was started on anti-coagulation (rivaroxaban 20 mg once daily) and bisoprolol 2.5 mg once daily. Later on, digoxin was added as the patient continued to have a fast ventricular response. Chemotherapy was changed to RCVP (Rituximab, Cyclophosphamide, Vincristine, and Prednisolone) protocol, and is currently post 3 cycles with a good clinical response and is planned for repeat imaging. He continues to be in AF and is under follow-up by cardiology.

### Case 3. Af diagnosis post bendamustine single agent in a patient with relapsed cHL

A 76-year-old woman, with hypothyroidism, who was diagnosed with cHL 8 years back (received 6 cycles of ABVD (doxorubicin, bleomycin, vinblastine, dacarbazine), was found to have biopsy proven relapse and received 10 cycles of brentuximab as salvage therapy. In spite of an initial good partial metabolic response, she had progressive disease on repeat PET-CT scan. Nivolumab and pembrolizumab were not available. She was therefore prescribed bendamustine as a single agent for the treatment of her symptomatic advanced stage disease.

One day after the first dose of bendamustine 90 mg/m^2^, she developed palpitations and dizziness, and had an irregular pulse. ECG showed new onset AF. 2D-Echo showed a dilated left atrium, mild-moderate mitral regurgitation, mild tricuspid regurgitation, basal infer-septal hypokinesia and an EF of 45–50%. CHADSVASC score was 3. She was started on dabigatran 150 mg, twice daily and bisoprolol 5 mg once daily.

Because of active symptomatic disease, one month later, the patient was given a second cycle of bendamustine, followed by the third cycle a month later. Ten days after the third cycle, the patient presented to the hospital with a productive cough and shortness of breath. She denied any chest pain or palpitations. Her ECG showed episodes of AF with HR of 158 to 162 bpm. She was seen by the cardiology team, and had a repeat ECHO which showed an EF of 40–50%. Digoxin was added to her bisoprolol with good rate control, and anti-coagulation was continued. Unfortunately, the patient had progressive disease, developed severe chest infection and died few months later.

## Discussion

AF among patients with cancer is associated with increased morbidity and mortality.^7^ It can also affect the choices of anti-cancer agents. Arrhythmias, and particularly AF are possible complications of several cancer therapies. Most drug-related AF data are derived from small and non-randomized studies. Published data, for the most part, is missing critical information on whether the reported patients had AF before cancer diagnosis and before treatment initiation or not. This is important information to establish a causality relationship between cancer treatment and AF occurrence. Diagnosing drug-induced AF is difficult as it lacks a clear biomarker.^2,7^ To the best of our knowledge, our report is the first to provide detailed reports of patients with lymphoma, and who developed AF within 24–48 h of their exposure to bendamustine without any clear underlying cause and without prior history of AF. Although two of the patients had existing risk factors to get AF (particularly age), we believe that bendamustine is the potential cause, as the onset of AF symptoms among the three patients was 1–2 days after receiving bendamustine.

To assess the correlation between the AF and bendamustine, the Naranjo Nomogram was applied, which is considered one of the most accepted tools for assessing the causality of adverse drug reactions with the suspected drug. The correlation came as “probable” with scores between 5 and 8. Points that may be distributed to be highly certain come from the presence of previous reliable sources listing this reaction (+ 1), the adverse event appearing after the drug was given (+ 2), confirmation of the event by objective evidence (+ 1), and the adverse reaction reappeared when the drug was re-administered (+2) with case 3. The points that may be up for debate are from the presence of alternative causes (− 1 to + 2). This is debatable as patients 1 and 2 were on rituximab, which has been reported in case reports as being associated with arrhythmias, including AF.

Several major trials involving bendamustine-containing regimens reported AF as a side effect of clinical concern with it's use. Examples are the SHINE trial, the ALLIANCE trial, and the HELIOS trial. The SHINE trial was a phase III trial conducted on 523 older patients with previously untreated mantle cell lymphoma (MCL). The study evaluated the outcome of adding Ibrutinib to the BR. It was a two-arm study where the patients enrolled to receive ibrutinib + BR or BR alone. Although the incidence of AF was higher in the ibrutinib group, placebo patients developed it (36 versus 17 cases, respectively).^4–6^ The ALLIANCE trial, on the other hand, was a randomized phase III trial conducted on 547 older patients with untreated CLL designed as a three arms study (arm1: BR, arm2: ibrutinib, arm3: ibrutinib/rituximab). Three percent (5/183) of patients in the BR group developed AF. The cumulative incidence of AF (grade 3 or higher) in arm 1 was 1.1%, 1.8%, 2.4%, and 3.5% at 6, 12, 24, and 36 months, respectively. However, arms 2 & 3 (ibrutinib-based) had a higher AF incidence and cumulative incidence of 3.1%, 4.5%, 6.2%, and 7.7% at 6, 12, 24, and 36 months, respectively.^5^ The HELIOS trial was was a phase III randomized, double-blind, and controlled trial on 578 patients with relapsed referactory CLL or small lymphocytic lymphoma (SLL) investigating the benefits of adding ibrutinib to the BR therapy. Grade 1–2 AF was reported in seven patients of the placebo group (BR alone), and 29 patients in the ibrutinib group.^6^ In all these trials, details on this in terms of timing and risk factors for AF are lacking.

Two of our cases were old patients (older than 70). Older age increases the risk of AF, and is considered one of the strongest non-modifiable risk factors. SHINE and ALLIANCE trials involved patients with a median age of 71 years old. Other large trials involving bendamustine use in a younger population (including the BRIGHT, GELTAMO, and GALLIUM trials), did not mention to report any case for AF. BRIGHT was a phase III randomized study that evaluated the safety and efficacy of BR therapy versus standard therapy (Rituximab, Cyclophosphamide, Doxorubicin, Vincristine, and Prednisolone (R-CHOP)/R-CVP) as a first-line treatment in 447 patients with indolent NHL or MCL. The median age of patients who were randomized to receive BR was 60 years. A comparable median age was reported in both GELTAMO and GALLIUM trials of 65 and 58–60 years, respectively. The GELTAMO trial was a phase II multicenter trial evaluating BR treatment as first line for MZL, while the GALLIUM trial was a phase III trial on 195 patients with previously untreated MZL who were randomized to receive obinutuzumab or rituximab combined with chemotherapy (with bendamustine, CHOP or CVP).^4,5,13–15^

Our second case was the oldest patient (older than 80 years old) and had additional multiple risk factors for AF such as HTN, diabetes, and IHD. However, our first case represents a middle-aged lady without any known comorbidities or risk factors for AF. The first and third cases received higher doses of bendamustine (90 mg/m^2^) among the three patients. The risk of AF could be higher with higher doses of bendamustine, even in younger patients without known comorbidities. Both cases 2 and 3 had hypothyroidism as a comorbidity. Patients with hyperthyroidism are at increased risk for developing AF. However, hypothyroidism, though associated with a higher incidence of cardiovascular events, it's influence on AF occurrence is undefined.^16^

All the previous trials that reported AF among patients receiving bendamustine are missing critical information as staged earlier. One essential piece of information is whether the patients had AF before cancer diagnosis and before treatment initiation or not. One exception is perhaps the HELIOS trial. It was clearly stated that some of the enrolled patients in both study arms had a history of AF or atrial flutter before treatment. As per the SRealCLL study, cardiac arrhythmias (including AF) are the second most common comorbidities in patients with CLL in Spain. This study included 558 patients, and 258 of them had this comorbidity either before or after receiving CLL treatment. 43.3% of the patients who had AF, were in the watch-and-wait phase. In this report, none of the three cases had a known history of AF before bendamustine initiation. Therefore, it may be reasonable to assume that AF occurrence was induced, at least in part, by bendamustine.^6,17^ Two of the three cases were patients with newly diagnosed lymphoma, who had not started on any treatment, and one of the cases had relapsed disease. One of the patients received 6 cycles of doxorubicin 8 years prior to the use of bendamustine but had a preserved EF at the time of relapse and at the time of diagnosis of AF. It is unlikely that other possible agent induced AF among these patients. Two patients had rituximab in combination with the bendamustine. Rituximab has been reported in case reports as being associated with arrythmias including AF.^18–21^ Some of these were reported in the setting of infusional reactions to rituximab. The aforementioned randomized trials that had bendamustine as part of the treatment regimens, included rituximab. In our patients, two had rituximab along with bendamustine. None of them had an infusional reaction. One of them had rituximab as part of next therapy and tolerated that well. Therefore, it is reasonable to assume that bendamustine is the most likely responsible agent.

To conclude, we believe that reporting rare and unusual side effects of commonly used medications is important. New-onset AF is mostly induced by bendamustine, though reported, has not been well described. Our report is limited to three patients. However, our paper is the first to describe this in detail among patients without prior documented history of AF, and with and without known risk factors for AF. Further studies are needed to confirm the relationship between AF and exposure to bendamustine and to identify the possible risk factors and responsible mechanisms.
